# Differentiating IDH status in human gliomas using machine learning and multiparametric MR/PET

**DOI:** 10.1186/s40644-021-00396-5

**Published:** 2021-03-10

**Authors:** Hiroyuki Tatekawa, Akifumi Hagiwara, Hiroyuki Uetani, Shadfar Bahri, Catalina Raymond, Albert Lai, Timothy F. Cloughesy, Phioanh L. Nghiemphu, Linda M. Liau, Whitney B. Pope, Noriko Salamon, Benjamin M. Ellingson

**Affiliations:** 1 UCLA Brain Tumor Imaging Laboratory (BTIL), Center for Computer Vision and Imaging Biomarkers, David Geffen School of Medicine, University of California Los Angeles, Los Angeles, USA; 2 Department of Radiological Science, David Geffen School of Medicine, University of California Los Angeles, Los Angeles, USA; 3 Department of Diagnostic and Interventional Radiology, Osaka City University Graduate School of Medicine, Osaka, Japan; 4 Department of Radiology, Juntendo University School of Medicine, Tokyo, Japan; 5 Department of Diagnostic Radiology, Faculty of Life Sciences, Kumamoto University, Kumamoto, Japan; 6 Department of Molecular and Medical Pharmacology, David Geffen School of Medicine, University of California Los Angeles, Los Angeles, USA; 7 UCLA Neuro-Oncology Program, David Geffen School of Medicine, University of California Los Angeles, Los Angeles, USA; 8 Department of Neurology, David Geffen School of Medicine, University of California Los Angeles, Los Angeles, USA; 9 Department of Neurosurgery, David Geffen School of Medicine, University of California Los Angeles, Los Angeles, USA

**Keywords:** Machine learning, ^18^F-DOPA PET, MRI, IDH mutation, Clustering, Diffuse glioma

## Abstract

**Background:**

The purpose of this study was to develop a voxel-wise clustering method of multiparametric magnetic resonance imaging (MRI) and 3,4-dihydroxy-6-[^18^F]-fluoro-L-phenylalanine (FDOPA) positron emission tomography (PET) images using an unsupervised, two-level clustering approach followed by support vector machine in order to classify the isocitrate dehydrogenase (IDH) status of gliomas.

**Methods:**

Sixty-two treatment-naïve glioma patients who underwent FDOPA PET and MRI were retrospectively included. Contrast enhanced T1-weighted images, T2-weighted images, fluid-attenuated inversion recovery images, apparent diffusion coefficient maps, and relative cerebral blood volume maps, and FDOPA PET images were used for voxel-wise feature extraction. An unsupervised two-level clustering approach, including a self-organizing map followed by the K-means algorithm was used, and each class label was applied to the original images. The logarithmic ratio of labels in each class within tumor regions was applied to a support vector machine to differentiate IDH mutation status. The area under the curve (AUC) of receiver operating characteristic curves, accuracy, and F1-socore were calculated and used as metrics for performance.

**Results:**

The associations of multiparametric imaging values in each cluster were successfully visualized. Multiparametric images with 16-class clustering revealed the highest classification performance to differentiate IDH status with the AUC, accuracy, and F1-score of 0.81, 0.76, and 0.76, respectively.

**Conclusions:**

Machine learning using an unsupervised two-level clustering approach followed by a support vector machine classified the IDH mutation status of gliomas, and visualized voxel-wise features from multiparametric MRI and FDOPA PET images. Unsupervised clustered features may improve the understanding of prioritizing multiparametric imaging for classifying IDH status.

**Supplementary Information:**

The online version contains supplementary material available at 10.1186/s40644-021-00396-5.

## Background

The World Health Organization (WHO) classification of Tumors of the Central Nervous System was revised in 2016, and the molecular status, such as isocitrate dehydrogenase (IDH) gene mutation and chromosomal 1p/19q co-deletion, was integrated to diagnose diffuse gliomas [1]. Because prognosis and patient management differ depending on the IDH mutation status, predicting the genotype before surgery has become more important in clinical situations. Several studies have evaluated magnetic resonance imaging (MRI) and amino acid positron emission tomography (PET) images to determine IDH status, revealing specific imaging features in relation to different IDH statuses [2].

Radiomics, including texture analysis, is a well-known and widely-used method for image feature extraction using a machine learning technique [3, 4]. Thousands of radiomic features can be quantified, and parts of them are selected through computational algorithms to yield quantitative imaging biomarkers that characterize intra-tumoral specific features. Many radiomics studies on diffuse gliomas assess the classification performance of the molecular status using multiparametric MRI sequences [5–9]. Amino acid PET, such as 3,4-dihydroxy-6-[^18^F]-fluoro-L-phenylalanine (FDOPA) and O-(2-[^18^F] fluoroethyl)-L-tyrosine (FET), provides metabolic information to complement MRI scan-derived information, and was also integrated into radiomics approaches [10]. These machine learning studies separately extracted texture features from different sequences, and then combined the features independently to train/validate the classifier. Thus, they did not account for the voxel-wise association of different sequences. Furthermore, most extracted features were intuitively difficult to understand from a clinical standpoint.

A two-level clustering approach, which was introduced by Inano et al. [11, 12], may provide a new insight in machine learning to help understand the specific imaging associations. This consists of an unsupervised clustering method with a self-organizing map (SOM) followed by a K-means and effectively differentiated glioma grades. SOM is a well-known type of neural-network unsupervised learning method that simplifies multiparametric features to clusters, defined as ‘protoclusters’. These protoclusters are then classified into the expected number of clusters by a K-means algorithm. This two-level clustering approach has the following three important benefits compared with the standard K-means algorithm. First, although the K-means algorithm is very sensitive to outliers, protoclusters of SOM are local averages of the input vectors and outliers can be eased; hence, they are more robust to outliers than conventional K-means. Second, this method can reduce computational costs. Third, the two-dimensional arrangement of the larger protoclusters by SOM can be easily visualized, thus providing useful information on the features of interest.

The purpose of this study was to develop a voxel-based clustering method of multiparametric MRI and FDOPA PET images using an unsupervised, two-level clustering approach followed by support vector machine (SVM) to classify the IDH mutation status of gliomas. We hypothesize that this approach may help visualize the association of multiparametric imaging metrics, as well as differentiate IDH status.

## Methods

### Patient selection

A total of 69 patients with newly diagnosed, treatment-naïve, and histologically confirmed diffuse gliomas who underwent FDOPA PET and MRI scans between 2010 and 2020 were retrospectively selected. By biopsy or surgical resection, all patients were diagnosed with WHO grade II, III, or IV diffuse gliomas according to the 2007 or 2016 WHO classification of Tumors of the CNS [1, 13]. Exclusion criteria were as follows: 1) sever image artifacts (*n* = 2); 2) absence of apparent diffusion coefficient (ADC) maps with b value = 1000 s/mm^2^ (*n* = 5). Finally, 62 patients who fulfilled the eligibility criteria were included, and further classified by IDH mutation status, which was determined by genomic sequencing analysis using polymerase chain reaction, and 1p19q codeletion status, which was assessed using fluorescence in situ hybridization. The MRI were obtained within 2-month interval of the corresponding PET scans for all patients. No patients underwent a stereotactic biopsy before FDOPA PET or MRI scan. The study was approved by the institutional review board at UCLA, and all subjects signed an informed consent form. Most subjects were included in two previous studies: evaluation of voxel-wise imaging correlations between FDOPA PET and MRI [14], and evaluation of the utilities of hypermetabolic regions of interest (ROIs) [15].

### MRI acquisition

Anatomical MRIs consisted of standard pre- and post-contrast T1-weighted images (T1WI) at 2D axial turbo spin echo (3 mm slice thickness and no interslice gap) or 3D inversion-prepared gradient echo images (1.0–1.2 mm isotropic voxel size) using a 1.5-T or 3-T clinical MRI scanner [16]. T2-weighted images (T2WI) and T2-weighted fluid-attenuated inversion recovery (FLAIR) images (3 mm slice thickness with no interslice gap) were also acquired.

Diffusion weighted imaging (DWI) was acquired for seven subjects (3 mm slice thickness and no interslice gap). From the acquired DWI with b = 1000 s/mm^2^ and b = 0 s/mm^2^ images, ADC maps were calculated. Diffusion tensor imaging (DTI) data (2–3 mm slice thickness with no interslice gap) was collected for 55 subjects for whom conventional DWI was not obtained. The DTI was acquired with 12–64 equidistant diffusion-sensitizing directions with b = 1000 s/mm^2^, as well as a single b = 0 s/mm^2^ image. After motion and eddy-correction, mean diffusivity maps were calculated and used as estimates of ADC values using FSL software (*eddy* and *dtifit;* FMRIB, Oxford, UK; http://www.fmrib.ox.ac.uk/fsl/).

For dynamic susceptibility contrast (DSC) MRI, a total dose of 0.1 mmol/kg of gadolinium contrast material (Gadavist or Magnevist; Bayer HealthCare Pharmaceuticals, Wayne, NJ, USA) was administered. A total dose of 0.025 mmol/kg was used for the preload dosage to mitigate T1-based leakage contamination, and the remaining 0.075 mmol/kg were used for dynamic bolus administration. Between the preload dose and the start of baseline DSC-MRI, a 2-min gap was incorporated. The DSC-MRI (5 mm slice thickness and no interslice gap) was acquired with 10–25 baseline acquisitions before contrast agent injection at 120 timepoints. Using FSL software (*mcflirt*), dynamic time-series images were motion-corrected. After applying a bidirectional contrast agent leakage-correction algorithm [17], relative cerebral blood volume (rCBV) maps were subsequently computed by dividing each voxel of rCBV maps by the median value of 6–10 ROIs lain on the contralateral normal-appearing white matter.

Summary of MRI acquisition parameters is shown in Supplemental Table 1.

### FDOPA PET image acquisition

A full-ring PET/computed tomography (CT) scanner (ECAT-HR; Siemens, Knoxville, TN, USA) was used after the subjects fasted for more than 4 h to obtain FDOPA PET images. For attenuation correction, a CT scan (120 kV) was acquired before the PET scan. FDOPA was synthesized and injected intravenously [18, 19]. Three-dimensional FDOPA emission data were obtained for 30 min. To obtain 20-min static FDOPA images after reconstruction, the data were integrated between 10 and 30 min following the injection. FDOPA PET images were then reconstructed using an ordered-subset expectation maximization iterative reconstruction algorithm (6-iteration and 8-subset) [20, 21]. A Gaussian filter was then applied with a full width at half maximum of 4 mm. The resulting voxels were 1.34 × 1.34 × 3 mm for FDOPA PET images. The standardized uptake value (SUV) maps were calculated based on the radioactive activity of FDOPA divided by the decay-corrected injected dose per body mass.

### Postprocessing and signal intensity/SUV normalization

All MRI (T2WI, T2-weighted FLAIR images, rCBV maps, and ADC maps) and FDOPA PET images were registered to the post-contrast enhanced (CE) T1WI for each patient using a six-degree-of-freedom rigid transformation with a mutual information cost function using FSL software (*flirt*). A tumor ROI was segmented based on the hyperintense regions on whole brain T2-weighted FLAIR images for supervised evaluation by a single board-certificated neuroradiologist (H.T. with 12 years of clinical experience and 2 years of segmentation experience) with the Analysis of Functional NeuroImages software (NIMH Scientific and Statistical Computing Core; Bethesda, MD, USA; https://afni.nimh.nih.gov). A semi-automatic procedure was employed in which a large ROI was drawn over a FLAIR hyperintensity region and intensity thresholds were chosen for each patient to extract only FLAIR hyperintense regions [22]. In this study, six images, including CE-T1WI, T2WI, FLAIR images, ADC maps, rCBV maps, and FDOPA PET images, were used for machine learning. For signal intensity/SUV normalization, values ranging between 0 to 75 percentiles + 1.5 × interquartile range were assigned to a range between 0 to 1, and outliers were not excluded.

### Unsupervised two-level clustering approach

**Fig. 1:**
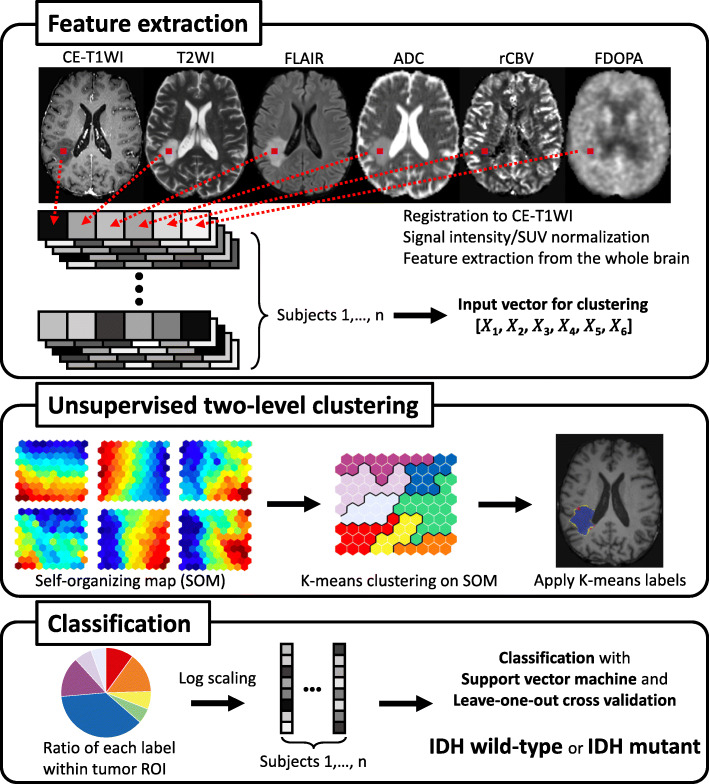
Simplified graphical overview of the processing

An overview of the following processing is shown in Fig. 1. By referring to the previous study about the analysis methods, including voxel size of feature extraction [11], features for unsupervised clustering were extracted from voxels on the six parameters of normalized original images every 64 (4 × 4 × 4) voxels within the binary whole brain mask image obtained with FSL’s brain extraction tool (*bet*). The extracted features from six different images of all subjects were stacked and used as input vectors (dimension: 6 × 1,520,745) for voxel-based clustering. A two-level clustering approach was applied using a batch-learning SOM and the K-means algorithm for unsupervised clustering [11]. A large number of input vectors was clustered into a much larger than expected number of protoclusters. Next, the protoclusters were classified into the expected number of clusters by a K-means algorithm using the weighted vectors of each protocluster. According to a previous study [11], we chose the numbers of K-class with K = 4, 6, 8, 10, 12, 16, 20. After unsupervised clustering by SOM followed by the K-means, 400 (20 × 20) protoclusters with K-class label information were generated. The label information of the nearest protocluster was assigned to each voxel on the six intensity-normalized original images within the tumor ROIs. To evaluate the ratios of labels for each K-class within tumor ROI, the common logarithmic value of the ratio was calculated by the formula: log_10_ (*p* + 10^− 2^), where *p* is a ratio of each label (%). Then the ratios of each K-class label for all subjects were applied as input features (dimension: K-class × 62 [subjects]) to the following SVM classification. We implemented this two-level clustering algorithm using MATLAB software (R2019b; MathWorks, Natick, MA, USA).

### Classification using SVM

By applying the ratios of each K-class label as extracted features, which were calculated by the two-level clustering approach of six different images, a linear kernel SVM was chosen as a classifier to discriminate between IDH wild-type and mutant gliomas, and the hyperparameter (C) of the linear kernel SVM with a two-step grid search technique was optimized, as previously described [11]. A leave-one-out cross validation (LOOCV) strategy was carried out to assess the classification performance that is widely used in machine learning, allowing us to use most of the data for training. The decision function derived from the training datasets was used to classify or calculate a decision value for the test subject. After the LOOCV, the area under the curve (AUC) of receiver operating characteristic (ROC) curves, accuracy, sensitivity, specificity, precision, recall, and F1-score were calculated. Additionally, the patients’ age was included to the SVM analysis to evaluate differentiation performances. We used Python (version 3.6.4) and scikit-learn library (version 0.23.1; https://scikit-learn.org/stable/) to implement a linear kernel SVM, LOOCV strategy, and the following bootstrap technique.

### Statistical analysis

To determine if the classification performances were significantly different among the different K-classes (K = 4, 6, 8, 10, 12, 16, 20), we performed SVM classification in each K-class 100 times using a bootstrap technique, and then analyzed the differences by a one-way analysis of variance (ANOVA) followed by Tukey’s multiple comparison tests. To compare the log-ratio values of each label in the K-class with the best classification performance between IDH wild-type and mutant, Mann–Whitney *U* test with Benjamini-Hochberg method for the multiple comparison corrections was used. Statistical significance was defined as *P* < 0.05.

## Results

**Fig. 2:**
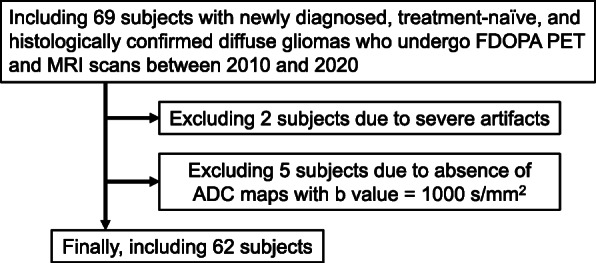
Patient selection flow-chart

This study included 62 treatment-naïve glioma patients (23 females) of a mean age of 53.0 years at the time of the PET examination (Table 1 and Supplement Table 2). A patient selection flow-chart is shown in Fig. 2. According to the 2007/2016 WHO criteria, 13 gliomas were grade IV, 21 were grade III, and 28 were grade II; 33 gliomas were IDH wild-type, and 29 were IDH mutant (17 were 1p/19q non-codeleted and 12 were 1p/19q codeleted).

**Table 1:** Patient demographics and molecular information

No. of patients	62
No. of female patients	23 (37%)
Age ± standard deviation (year)	53.0 ± 14.8
WHO classification grade	
II	28 (45%)
III	21 (34%)
IV	13 (21%)
IDH mutation status	
Wild-type	33 (53%)
Mutant	29 (47%)
1p/19q codeletion status for IDH mutant	
1p/19q non-codeleted	17 (28%)
1p/19q codeleted	12 (19%)

*IDH* isocitrate dehydrogenase

To determine the number of K-classes which showed the best prediction performance for IDH mutation, AUC, accuracy, and F1-score were compared among different K-classes using a bootstrap method. The 16-class clustering showed the highest AUC, accuracy, and F1-score, which were significantly higher than the other K-classes (K = 4, 6, 8, 10, 12), except for the 20-class clustering (Supplemental Fig. 1). To understand the following results clearly, we primarily showed the results of the K = 16-class for the following analyses. The prediction performance for all K-classes except K = 16 are summarized in Supplemental Table 3.

**Fig. 3:**
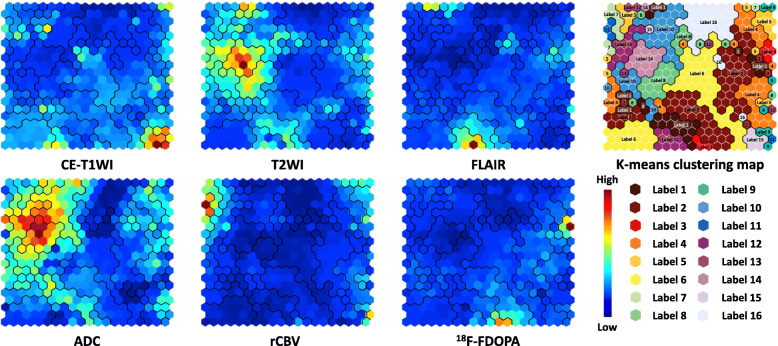
Component planes with SOM for each imaging parameter ranging from blue to red according to each value. Red means a high weight. The inter-class borderlines obtained by K-means clustering with K = 16 are shown on the SOM component planes as black lines between the nodes. Detailed profiles can be seen on the K-means clustering map (from label 1 to 16)

**Fig. 4:**
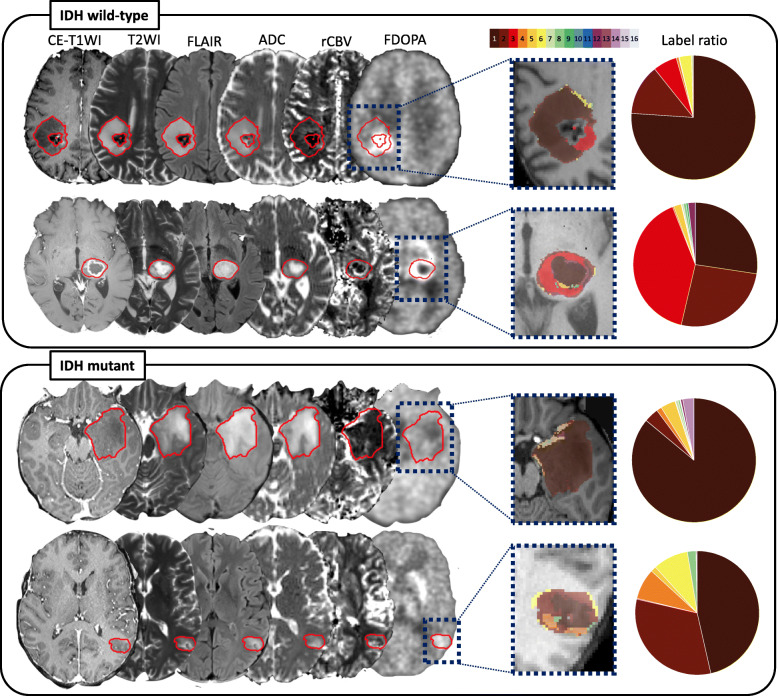
Representative cases of IDH wild-type and mutant gliomas with 16-class clustering that shows the highest classification performance. The CE-T1WI, T2WI, and FLAIR image, and ADC, rCBV, and FDOPA maps are shown for each patient. Each color within the tumor ROIs corresponds to each label in the 16-color bar. The ratios of each label are shown in pie chart. The voxels of the label 3 (red) can be seen frequently for the IDH wild-type gliomas

The component planes of the six variables from CE-T1WI, T2WI, FLAIR, ADC, rCBV, and FDOPA by the SOM analysis showed the information of each sequence in each map unit as well as the associations between the protoclusters and each image (Fig. 3). The component planes of 6 variables differed from each other, but those of T2WI and ADC showed similar mapping. Next, the protoclusters were successfully classified into 16 labels by K-means (K = 16), each label of which was applied to the tumor ROIs. Figure 4 shows representative cases of IDH wild-type and mutant gliomas. In these cases, the voxels of labels 1 and 2 occupied a majority of tumor ROIs for both IDH wild-type and mutant gliomas, while the voxels of label 3 can be seen more frequently for the IDH wild-type gliomas than mutant gliomas.

**Fig. 5:**
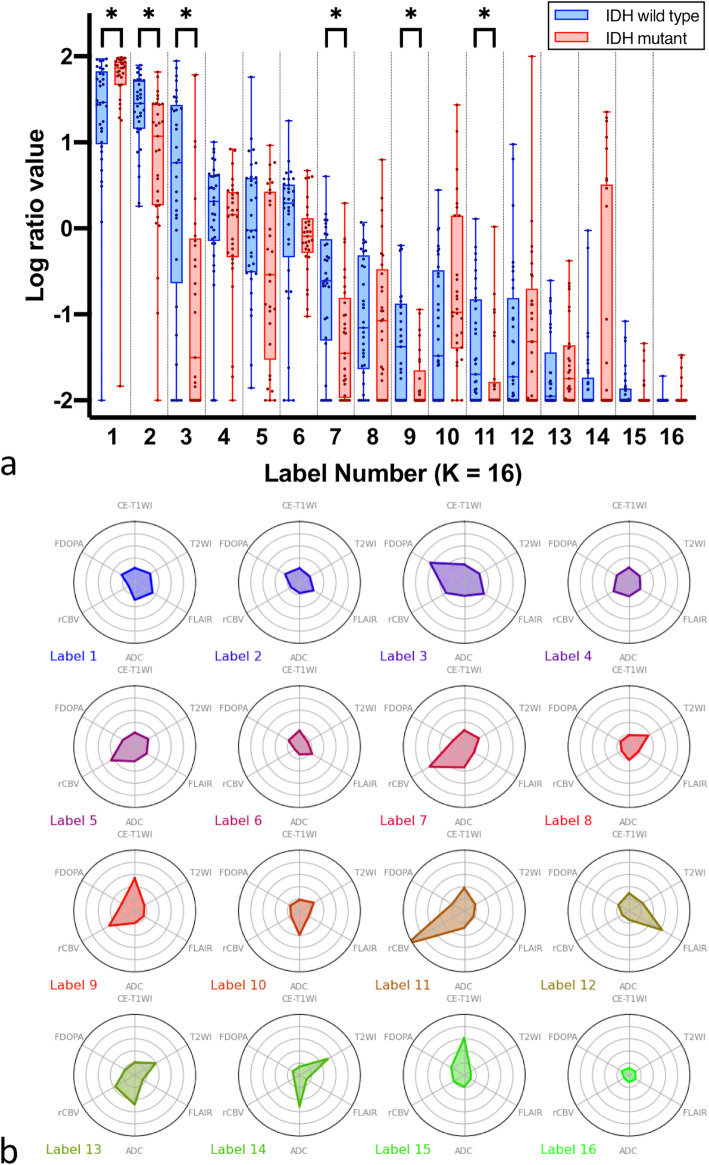
**a**) Box-whisker plots and **b**) radar charts of each label by 16-class clustering. **a** The box-whisker plots showing median and interquartile range for log-ratio values. * shows a significant difference. **b** Radar charts of six variables (CE-T1WI, T2WI, FLAIR, ADC, rCBV, and FDOPA PET) in each label by 16-class clustering

As for the classification performance using SVM with LOOCV in K = 16, the AUC, accuracy, sensitivity, specificity, precision, recall, and F1-score were 0.81, 0.76, 0.75, 0.82, 0.78, 0.81, and 0.76, respectively. When age was included in the SVM analysis, the AUC, accuracy, sensitivity, specificity, precision, recall, and F1-score were 0.87, 0.84, 0.91, 0.76, 0.81, 0.91, and 0.86, respectively. The log-ratio values of each label in the K = 16 class were compared between IDH wild-type and mutant gliomas (Fig. 5a). The values of the labels 2, 3, 7, 9, and 11 were significantly higher in IDH wild-type than in mutant gliomas (*P* = 0.001, < 0.001, 0.006, 0.006, and 0.007, respectively), whereas the value of the label 1 was significantly higher in IDH mutant than in wild-type gliomas (*P* = 0.001). The radar charts of the individual normalized values of the six images for each label in the K = 16 class are shown in Fig. 5b. The chart patterns of label 3, which showed significantly higher log-ratio values in IDH wild-type than in mutant, consisted of high FDOPA values. The labels 7 and 11, which were also significantly higher in IDH wild-type than in mutant, had high rCBV values.

## Discussion

This study developed a voxel-based clustering method of multiparametric images, including CE-T1WI, T2WI, FLAIR, ADC, rCBV, and FDOPA PET, using an unsupervised two-level clustering approach, and evaluated the classification ability of IDH wild-type and mutant gliomas using SVM with LOOCV. This method enabled the visualization of the association of imaging values in each cluster. Multiparametric images with 16-class clustering showed the highest classification performance to differentiate IDH status with the AUC, accuracy, and F1-score of 0.81, 0.76, and 0.76, respectively. Further, when age was included to the SVM, the AUC, accuracy, and F1-score improved to 0.87, 0.84, and 0.86, respectively.

Several previous studies have identified specific features of gliomas in relation to IDH mutation status. IDH wild-type gliomas were reported to have a higher likelihood of contrast enhancement, higher rCBV, lower ADC value than IDH mutant gliomas, while a well-defined border and T2–FLAIR mismatch sign were specific features of IDH mutant gliomas [2]. A meta-analysis revealed a summary sensitivity and specificity of 86 and 87%, respectively, to differentiate IDH mutation status, which was similar to or slightly superior to our results [2]. Meanwhile, IDH wild-type gliomas, especially glioblastomas, grew into the periventricular white matter adjacent to the subventricular zone [23, 24], whereas more than half of IDH mutant gliomas were localized in the frontal lobe [25]. However, these locational features were overlapping and may not be useful for differentiation purposes.

Previous machine learning studies generally performed a texture analysis to differentiate IDH status. Zhang et al. [7] and Zhou et al. [8] reported a higher accuracy (0.89) and AUC (0.92), respectively, to differentiate IDH status, as compared to our study. However, these studies included patient age as well as multiparametric MRI features as variables in all analyses, with the variable age offering the highest predictive value. Patients with IDH wild-type gliomas were known to be significantly older than patients with IDH mutant gliomas [26]; therefore, patient age may largely affect the classification performance, and the differentiation performance using imaging features alone cannot be compared with the current study. Li et al. [5] used imbalanced data set and reported that multiparametric MRI could predict IDH status with an F1-score of 0.78, which was similar to our study.

When comparing with previous machine learning studies using amino acid PET, Lohmann et al. [27] reported that combining texture features of FET PET with standard PET parameters differentiated IDH status with high accuracy (0.93) [27]. Haubold et al. reported that a combination of texture features of FET PET with multiparametric MRI classified IDH status with an AUC of 0.88 [10]. These studies using only imaging features and the classification performances are superior to our results. However, these cohorts used imbalanced data with a larger number of IDH wild-type gliomas, and did not calculate F1-scores. For the evaluation of classification performance, accuracy and ROC can be used when the class distributions are similar, while F1-scores should be used when there are imbalanced classes. Thus, the true classification performance of these studies remains unclear, and cannot be directly compared with our results.

Although differentiation performances were similar to previous studies, this study can help visualize which imaging parameters play important roles in classifying IDH status using unsupervised clustered features. This is the core strength of this study and may help understand the priority of multiparametric images in clinical situations. The ratio of label 3 in 16-class clustering was significantly higher in IDH wild-type than mutant gliomas. The radar charts of six variables showed high FDOPA values in label 3, which was mapped to FDOPA hypermetabolic areas on the original images. Higher ratios of label 3 in IDH wild-type than mutant gliomas may reflect larger hypermetabolic volume in IDH wild-type gliomas. This may be partly due to the different ratios of included subjects because all grade IV glioblastomas were IDH wild-type, and glioblastomas tend to have a large hypermetabolic volume [28]. Indeed, when excluding FDOPA in this machine learning analysis (detailed data not shown), the specificity and recall decreased to 0.75 and 0.76, respectively, although other metrics retained similar values. These results suggested that parameters of FDOPA PET have an additional value in differentiating IDH status. Similarly, the ratios of labels 7 and 11, which showed high rCBV values on the radar chart, were also significantly higher in IDH wild-type than mutant gliomas, reflecting larger high-rCBV spots in IDH wild-type gliomas. This is consistent with a previous study showing relatively high rCBV in IDH wild-type gliomas [2]. We believe that these clustering features allow for the improved establishment of feature extraction priorities for IDH wild-type gliomas since the associations are complex.

In contrast, the ratios of labels 1 and 2 in a 16-class occupied a large portion of a tumor in both IDH statuses, although the ratios significantly differed between IDH wild-type and mutant gliomas. However, the radar charts revealed non-specific small values in all parameters, suggesting that the majority of the components within tumor regions were of limited use for genotype classification.

There are specific limitations to this study that should be addressed. First, because the population of either IDH mutant 1p/19q non-codeleted (17/62) or codeleted gliomas (12/62) was not large, the classification performance to differentiate either of them from other subtypes or that of 3-class differentiation was not reliable (differentiation of IDH mutant 1p/19q non-codeleted, F1-score 0.69; differentiation of IDH mutant 1p/19q non-codeleted, F1-score 0.12; 3-class differentiation, F1-score 0.48; other detailed data not shown); hence, we combined these two groups as IDH mutant gliomas (29/62). However, gliomas with different 1p/19q codeletion statuses must have specific imaging/pathological features, such as relatively higher rCBV and FDOPA uptake in IDH mutant 1p/19q codeleted gliomas than in non-codeleted gliomas [29, 30]; hence, when using a much larger cohort, our method may help visualize specific imaging features for IDH mutant 1p/19q codeleted and non-codeleted gliomas. Second, although this study used LOOCV for predicting molecular status, another independent cohort is required to validate our classification performance. Third, due to the retrospective nature of this study, the acquisition parameters and scanners of MRI were standardized [16] but not identical across patients. For example, conventional DWI was obtained when DTI was not available, and mean diffusivity maps were used as estimates of ADC values instead of conventional ADC maps. However, the differences in sequences were mitigated by normalizing each parameter. In fact, the diversity of imaging acquisition parameters may have actually helped generalize the classification performance across a variety of acquisition parameters, as would be expected in a multicenter study.

## Conclusion

Machine learning using an unsupervised two-level clustering approach can be used to extract and visualize voxel-based imaging features from multiparametric MRI and FDOPA PET images and classify IDH mutation status using SVM with LOOCV. Unsupervised clustered features have revealed voxel-wise imaging associations, and may improve the understanding of the priority of multiparametric imaging features for classifying IDH mutation status, which may help predict molecular status before surgery.

## Supplementary Information

**Additional file 1: Supplemental Fig. 1.** The AUC, accuracy, and F1-score to classify IDH mutation status for different K number (K = 4, 6, 8, 10, 12, 16, 20) using 100 times bootstrap technique.

**Additional file 2: Supplemental Table 1.** Machine information and acquisition parameters.

**Additional file 3: Supplemental Table 2.** Detailed patient demographics and molecular information.

**Additional file 4: Supplemental Table 3.** Prediction performances of other K-class.

## Data Availability

The datasets used and/or analysed during the current study are available from the corresponding author on reasonable request.

## Abbreviations

WHO: World Health Organization
IDH: Isocitrate dehydrogenase
MRI: Magnetic resonance imaging
PET: Positron emission tomography
FDOPA: 3,4-dihydroxy-6-[18F]-fluoro-L-phenylalanine
FET: O-(2-[18F] fluoroethyl)-L-tyrosine
SOM: Self-organizing map
SVM: Support vector machine
ROI: Region of interest
T1WI: T1-weighted images
T2WI: T2-weighted images
FLAIR: Fluid-attenuated inversion recovery
DWI: Diffusion weighted image
ADC: Apparent diffusion coefficient
DTI: Diffusion tensor imaging
DSC: Dynamic susceptibility contrast
CT: Computed tomography
SUV: Standardized uptake value
CE: Post-contrast enhanced
LOOCV: Leave-one-out cross validation
AUC: Area under the curve
ROC: Receiver operating characteristic;
ANOVA: Analysis of variance
