# Supplementary material for: Differentiating IDH status in human gliomas using machine learning and multiparametric MR/PET
Source: Cancer Imaging. 2021 Mar 10;21:27. doi: 10.1186/s40644-021-00396-5 (PMC7944911; doi:10.1186/s40644-021-00396-5)
Supplement: Supplementary file 1 — Additional file 1: Supplemental Fig. 1. The AUC, accuracy, and F1-score to classify IDH mutation status for different K number (K = 4, 6, 8, 10, 12, 16, 20) using 100 times bootstrap technique. [file 40644_2021_396_MOESM1_ESM.docx]

**Supplemental Figure**

**Supplemental Fig. 1** a) The AUC, b) accuracy, and c) F1-score to classify IDH mutation status for different K number (K = 4, 6, 8, 10, 12, 16, 20) using 100 times bootstrap technique. Bars indicate mean values with 95% confidence interval. One-way ANOVA showed significant differences in all analyses (all *Ps* < 0.001). The 16-class clustering shows the highest AUC, accuracy, and F1-score, and significantly higher than the other K numbers except the 20-class clustering.
