# Supplementary material for: Differentiating IDH status in human gliomas using machine learning and multiparametric MR/PET
Source: Cancer Imaging. 2021 Mar 10;21:27. doi: 10.1186/s40644-021-00396-5 (PMC7944911; doi:10.1186/s40644-021-00396-5)
Supplement: Supplementary file 2 — Additional file 2: Supplemental Table 1. Machine information and acquisition parameters. [file 40644_2021_396_MOESM2_ESM.docx]

| **Supplemental Table 1** Machine information and acquisition parameters | | | | | | |  |
| --- | --- | --- | --- | --- | --- | --- | --- |
| Magnetic field strength | |  | 3 Tesla | 54 |  | 1.5 Tesla | 8 |
| Machine name |  |  | Prisma Fit | 11 |  | Avanto | 6 |
|  |  |  | Trio Tim | 18 |  | Genesis Signa | 2 |
|  |  |  | Skyra | 21 |  |  |  |
|  |  |  | Verio | 1 |  |  |  |
|  |  |  | MAGNETOM Vida | 3 |  |  |  |
|  |  | N | TR (ms) | TE (ms) | TI (ms) | Flip Angle (°) | Slice thickness (mm) |
| Post contrast T1-weighted images | MPRAGE | 59 | 1580–2110 | 2.5–4.6 | 900–1100 | 9–15 | 0.9–1.0 |
|  | SE | 3 | 433–522 | 2.5–9.0 |  | 90 | 3.0 |
| T2-weighted images |  | 62 | 1960–8570 | 86–121 |  | 120–160 | 3.0 |
| FLAIR images |  | 62 | 7080–15130 | 81–120 | 2400–2500 | 103–160 | 3.0 |
| DTI/DWI | DTI | 55 | 5500–11700 | 66–100 |  | 90 | 2.0 |
|  | DWI | 7 | 4500–10000 | 93–108 |  | 90 | 3.0 |
| Perfusion images |  | 62 | 1250–1293 | 21–23 |  | 60 | 5.0 |
