# Supplementary material for: Differentiating IDH status in human gliomas using machine learning and multiparametric MR/PET
Source: Cancer Imaging. 2021 Mar 10;21:27. doi: 10.1186/s40644-021-00396-5 (PMC7944911; doi:10.1186/s40644-021-00396-5)
Supplement: Supplementary file 3 — Additional file 3: Supplemental Table 2. Detailed patient demographics and molecular information. [file 40644_2021_396_MOESM3_ESM.docx]

| **Supplemental Table 2** Detailed patient demographics and molecular information | | | | | |
| --- | --- | --- | --- | --- | --- |
| ID | Sex | Age | WHO grade | IDH mutation status | 1p/19q codeletion status |
| 1 | Male | 76 | IV | Wild-type | – |
| 2 | Female | 66 | IV | Wild-type | – |
| 3 | Female | 63 | IV | Wild-type | – |
| 4 | Male | 64 | IV | Wild-type | – |
| 5 | Male | 69 | IV | Wild-type | – |
| 6 | Male | 59 | IV | Wild-type | – |
| 7 | Male | 77 | IV | Wild-type | – |
| 8 | Male | 74 | IV | Wild-type | – |
| 9 | Female | 55 | IV | Wild-type | – |
| 10 | Male | 74 | IV | Wild-type | – |
| 11 | Male | 36 | IV | Wild-type | – |
| 12 | Male | 56 | IV | Wild-type | – |
| 13 | Female | 76 | IV | Wild-type | – |
| 14 | Male | 63 | III | Wild-type | – |
| 15 | Female | 63 | III | Wild-type | – |
| 16 | Female | 60 | III | Wild-type | – |
| 17 | Female | 68 | III | Wild-type | – |
| 18 | Female | 60 | III | Wild-type | – |
| 19 | Male | 48 | III | Wild-type | – |
| 20 | Female | 59 | III | Wild-type | – |
| 21 | Female | 61 | III | Wild-type | – |
| 22 | Male | 58 | III | Wild-type | – |
| 23 | Male | 53 | III | Wild-type | – |
| 24 | Male | 59 | III | Wild-type | – |
| 25 | Male | 68 | III | Wild-type | – |
| 26 | Male | 62 | III | Wild-type | – |
| 27 | Male | 64 | III | Wild-type | – |
| 28 | Female | 25 | III | Mutant | Non-codeleted |
| 29 | Male | 38 | III | Mutant | Non-codeleted |
| 30 | Male | 55 | III | Mutant | Non-codeleted |
| 31 | Female | 41 | III | Mutant | Non-codeleted |
| 32 | Female | 47 | III | Mutant | Non-codeleted |
| 33 | Male | 35 | III | Mutant | Non-codeleted |
| 34 | Male | 53 | III | Mutant | Codeleted |
| 35 | Male | 50 | II | Wild-type | – |
| 36 | Female | 63 | II | Wild-type | – |
| 37 | Male | 63 | II | Wild-type | – |
| 38 | Male | 61 | II | Wild-type | – |
| 39 | Female | 50 | II | Wild-type | – |
| 40 | Male | 69 | II | Wild-type | – |
| 41 | Male | 57 | II | Mutant | Non-codeleted |
| 42 | Male | 39 | II | Mutant | Non-codeleted |
| 43 | Female | 36 | II | Mutant | Non-codeleted |
| 44 | Male | 61 | II | Mutant | Non-codeleted |
| 45 | Male | 36 | II | Mutant | Non-codeleted |
| 46 | Male | 34 | II | Mutant | Non-codeleted |
| 47 | Female | 25 | II | Mutant | Non-codeleted |
| 48 | Female | 27 | II | Mutant | Non-codeleted |
| 49 | Male | 22 | II | Mutant | Non-codeleted |
| 50 | Male | 39 | II | Mutant | Non-codeleted |
| 51 | Male | 79 | II | Mutant | Non-codeleted |
| 52 | Female | 47 | II | Mutant | Codeleted |
| 53 | Female | 48 | II | Mutant | Codeleted |
| 54 | Male | 36 | II | Mutant | Codeleted |
| 55 | Female | 48 | II | Mutant | Codeleted |
| 56 | Female | 28 | II | Mutant | Codeleted |
| 57 | Male | 52 | II | Mutant | Codeleted |
| 58 | Male | 61 | II | Mutant | Codeleted |
| 59 | Male | 37 | II | Mutant | Codeleted |
| 60 | Male | 43 | II | Mutant | Codeleted |
| 61 | Male | 56 | II | Mutant | Codeleted |
| 62 | Female | 25 | II | Mutant | Codeleted |
