# Supplementary material for: Differentiating IDH status in human gliomas using machine learning and multiparametric MR/PET
Source: Cancer Imaging. 2021 Mar 10;21:27. doi: 10.1186/s40644-021-00396-5 (PMC7944911; doi:10.1186/s40644-021-00396-5)
Supplement: Supplementary file 4 — Additional file 4: Supplemental Table 3. Prediction performances of other K-class. [file 40644_2021_396_MOESM4_ESM.docx]

| **Supplemental Table 3** Prediction performances of other K-classes | | | | | |  |
| --- | --- | --- | --- | --- | --- | --- |
| Number of K-class | 4 | 6 | 8 | 10 | 12 | 20 |
| AUC | 0.80 | 0.82 | 0.87 | 0.85 | 0.87 | 0.82 |
| Accuracy | 0.79 | 0.81 | 0.82 | 0.84 | 0.82 | 0.73 |
| Sensitivity | 0.94 | 0.85 | 0.82 | 0.91 | 0.82 | 0.73 |
| Specificity | 0.62 | 0.76 | 0.83 | 0.76 | 0.83 | 0.72 |
| Precision | 0.74 | 0.80 | 0.84 | 0.81 | 0.84 | 0.75 |
| Recall | 0.94 | 0.84 | 0.82 | 0.91 | 0.82 | 0.73 |
| F1-score | 0.83 | 0.82 | 0.83 | 0.86 | 0.83 | 0.74 |
